# Bipolar disorder prevalence and psychotropic medication utilisation in Hong Kong and the United Kingdom

**DOI:** 10.1002/pds.5318

**Published:** 2021-07-08

**Authors:** Vanessa W.S. Ng, Kenneth K.C. Man, Le Gao, Esther W. Chan, Edwin H.M Lee, Joseph F Hayes, Ian C.K. Wong

**Affiliations:** 1 Centre for Safe Medication Practice and Research, Department of Pharmacology and Pharmacy, The University of Hong Kong, Hong Kong; 2 Research Department of Practice and Policy, School of Pharmacy, University College London, London, UK; 3 Department of Psychiatry, Li Ka Shing Faculty of Medicine, The University of Hong Kong, Hong Kong; 4 Laboratory of Data Discovery for Health (D^2^4H), Hong Kong Science Park, Hong Kong Special Administrative Region, China; 5 Division of Psychiatry, Faculty of Brain Sciences, University College London, London, UK

**Keywords:** Bipolar disorder, prevalence, prescribing trend, maintenance treatment, valproate, lithium

## Abstract

**Purpose:**

Bipolar disorder (BPD) is often an under-addressed mental disorder. Limited studies have investigated its epidemiology and drug utilisation in Hong Kong (HK) and the United Kingdom (UK) and thus local prescribing practices remain unclear. This study aimed to determine the prevalence of BPD and the prescribing of psychotropic medications as maintenance treatment from 2001-2018 in HK and the UK.

**Method:**

A retrospective study using the data from Clinical Data Analysis and Reporting System in HK and IQVIA Medical Research Data in the UK.

**Results:**

The prevalence of BPD diagnosis in HK and the UK more than doubled during study period. Some distinct changes in prescribing pattern over time were observed. Lithium use declined by 2.46% and 14.58% in HK and the UK, respectively. By 2018, patients were 4.6 times more likely to receive antidepressant monotherapy in the UK versus HK (15.62% vs 3.42%). In HK, 38.41% of women of childbearing age were prescribed valproate in 2018 compared with 8.46% in the UK.

**Conclusion:**

The prevalence of BPD diagnosis has been increasing in HK and the UK. The disparity in prescribing patterns of BPD maintenance treatment in two regions reflected three major issues in clinical practice: 1) under-prescribing of lithium in both regions, 2) antidepressant monotherapy in the UK and 3) overprescribing of valproate to women of childbearing age in HK. Review of current clinical treatment guidelines and regulations of prescribing practice by local clinicians should be immediately implemented to ensure the safe use of medications in patients with BPD.

## Introduction

Bipolar disorder (BPD) is a severe mental illness, which is often associated with higher risks of suicide, self-harm and mortality relative to the general population, creating a huge disease burden to patients and society ^1,2^. In 2017, approximately 46 million people worldwide were diagnosed with BPD with a 12-month prevalence varying from 0.3% to 1.2% by country^3,4^. Many studies have been published on the epidemiology of BPD using different diagnostic criteria and methodologies hence brings difficulties to compare the findings directly between studies ^5–7^. An interview study conducted in a Hong Kong (HK) community setting reported the 12-month prevalence of BPD-I and BPD-II were 1.4% and 0.5% respectively. A previous study using an electronic healthcare database from the United Kingdom (UK) reported the incidence of BPD in primary care setting fluctuated from 11.0 per 100000 person-year at risk (PYAR) in 2000 to 19.0 per 100000 PYAR in 2010 ^5,6^; however, limited studies investigated the changes in prevalence in recent years. On the other hand, there are significant advances in clinical knowledge about the effectiveness and safety of medication use in BPD, for instance, the superior efficacy of lithium in treating BPD compared to antipsychotics and antiepileptics, risk of birth defects and neurodevelopmental disorders in children due to maternal use of sodium valproate ^8–11^. Currently, different international clinical guidelines recommend lithium, valproate, antipsychotics (e.g. quetiapine, olanzapine) as the potential first line maintenance treatment, with add-on therapy of other antipsychotics, antiepileptics or antidepressants if mood stabilisation is not achieved^12–16^. Therefore, contemporary studies to evaluate the epidemiology of BPD and its pharmacotherapy are warranted. This study aimed to compare the prevalence of BPD and the prescribing trend of its treatment in HK and the UK, to examine the deviations between the clinical practice and current recommendations. Both regions have strong publicly funded universal healthcare systems and are highly subsidised by the governments but genetically (Chinese vs predominantly Caucasian) and culturally (Eastern vs Western) are hugely different. More importantly, the difference in policies also influences clinical prescribing practices. In the UK, NICE is the main driving force in promoting effective and safe prescribing in England and Wales, while the equivalent organisation does not exist in HK. Selecting these two regions will allow us to explore the differences and give us more insights.

## Methods

### Study design

We conducted a retrospective study to investigate the annual prevalence of BPD and the prescribing trends of psychotropic medications among the patients with BPD using the Clinical Data Analysis and Reporting System (CDARS) in HK and IQVIA Medical Research Data (IMRD) in the UK.

### Data sources

#### Clinical Data Analysis and Reporting System (CDARS)

CDARS is an electronic healthcare database developed by the Hospital Authority (HA), which is a statutory body managing all public hospital services in HK. The HA provides a wide range of public-funded healthcare services to all HK residents (>7.4 million), including hospitalisation, specialist and general outpatient clinics, accident and emergency services ^17^. Since 1995, the clinical data from patients who had ever used any of the healthcare services at HA, including demographics, diagnosis, medication dispensing records, outpatient and primary care clinics, emergency room attendances, laboratory tests and hospitalisation details, have been made available on CDARS for research and audit purposes ^18^. CDARS does not capture clinical data from the private healthcare sectors but a local study reported that approximately 88.5% of psychiatric patients utilised public mental health services under HA^19^. Therefore, the CDARS is likely to cover the majority of the HK population. All data were anonymised to maintain patients’ privacy and confidentiality. The accuracy of data has been well validated and extensively used in various epidemiological studies ^20–24^. The HA was set up by the British Government while it was the British Colony. Similar to the Bitish NHS system, HA system provides highly subsidied public healthcare system.

#### IQVIA Medical Research Data (IMRD)

IMRD, previously known as The Health Improvement Network, is a representative primary care database, which covers approximately 4.5% of the UK population. It collects patients’ electronic medical records from over 774 general practices and includes various information: demographics, diagnosis, prescriptions, laboratory tests, hospitalisations, any clinical measures at primary care and socioeconomic status measured by Townsend deprivation index. The validity and generalisability of IMRD database has been well recognised and widely applied in pharmacoepidemiology studies ^25–28^.

#### Study population

All individuals with at least one diagnosis record of BPD from database inception were identified from CDARS and IMRD between 1^st^ January 2001 and 31^st^ December 2018. Only diagnoses among patients aged 6 years and older were considered to avoid any misdiagnosis in young children. Diagnoses of BPD were identified using International Classification of Diseases, Ninth Revision, Clinical Modification (ICD-9-CM) codes in CDARS (296.0, 296.1, 296.4-296.8) and Read codes in IMRD ^29^.

#### Psychotropic medications

All prescriptions of study drugs between 1^st^ January 2001 and 31^st^ December 2018 were identified in CDARS and IMRD in individuals with BPD, after the first diagnosis record of BPD. British National Formulary was used for identification of study drugs, including lithium, antiepileptics (sodium valproate, carbamazepine, and lamotrigine only), antipsychotics, and antidepressants. Patients who received at least two prescriptions of any of the study drugs during the study period were included to ensure their concordance to the treatment prescribed. Only prescriptions from out-patient appointments and discharge prescriptions in CDARS were included to ensure the prescribed medications are for maintenance treatment.

#### Prevalence calculation

Since BPD is regarded as a lifelong mental disease and once patients are diagnosed with BPD, they are assumed to have BPD for the rest of their lives ^30^. Annual prevalence of BPD was calculated by dividing the total number of patients with a history of BPD diagnosis by the total mid-year population in that particular year in the respective regions. Patients with multiple diagnosis records within the same year were counted once only. It was stratified by sex and different age groups: 6-11 (children), 12-17 (adolescents), 18-30 (young adults), 31-44 (adults), 45-64 (middle-aged), 65-84 (retired), and ≥85 (the elderly). The mid-year population statistics were obtained from the Census and Statistics Department, HK and IMRD, the UK, respectively.

Annual prevalence of psychotropic medication prescribing was calculated for each psychotropic drug class according to a previous study of psychotropic drugs prescribing in HK ^31^. It was defined as the sum of treated patients who were prescribed any drugs from each drug class divided by annual prevalent cases of BPD during the study period. Patients with multiple prescriptions of each drug class within the same year were counted once only. We then further looked at the individual mood stabilisers (lithium, sodium valproate, carbamazepine, and lamotrigine) and antipsychotics, as well as valproate prescribing in women of childbearing age (15-49 years) using the same approach.

To better visualise the treatment trajectories of all women of childbearing age, they were categorized into three groups each year: 1) valproate users who received at least one valproate prescriptions within the particular year, 2) non-valproate users who did not receive any valproate prescriptions but other study medications throughout the year, and 3) untreated group who did not receive any of prescriptions of study medications within the particular year. A Sankey diagram was then generated to show the movement of female patients with childbearing potential who initiated, remained on valproate, or switched to other treatment alternatives over time during the observation period.

Annual prevalence was estimated with 95% confidence interval (CI) using Poisson regression and expressed as a percentage. Data analysis was conducted independently by V.N. and L.G. for quality control using Statistical Analysis System (version 9.4; SAS Institute) and R (version 3.5.3; R Core Team).

## Results

### Patient characteristics

A total of 17247 and 36187 patients were diagnosed with BPD from HK and the UK respectively from 2001 to 2018. The median age at the first diagnosis was 38.8 years among patients from HK and 43.65 years from the UK. More than 80% of patients in HK and the UK were treated by psychotropic medications during the study period. Treated patients had more neuropsychiatric comorbidities than untreated patients in both regions (Table 1).

### Prevalence of BPD

#### 1 HK

The overall prevalence of BPD increased almost 3-fold from 0.066% (95% CI 0.064-0.068) in 2001 to 0.184% (95% CI 0.180-0.187) in 2018. Female prevalence was higher than males. The rate of increase in males was similar to females (3.08-fold vs 2.90-fold) (Figure 1a). In 2018, the middle-aged group had the highest prevalence, rising rapidly from 0.071% (95% CI 0.067-0.075) in 2001 to 0.254% (95% CI 0.248-0.260) in 2018, with the largest rate of increase (3.58-fold) among all age groups (Figure 1b).

#### 2 UK

The prevalence of BPD doubled from 0.150% (95% CI 0.147-0.154) in 2001 to 0.357% (95% CI 0.350-0.363) in 2018. Females had a higher prevalence than males but the rate of increase was comparable in both genders (2.36-fold vs 2.40-fold) (Figure 1c). The prevalence of BPD was the highest in the middle-aged group, followed by adults and retired groups throughout the study period (Figure 1d).

### Psychotropic drug prescribing

#### 1 Drug classes

##### 1.1 HK

From 2001 to 2018, the most common drug class prescribed to patients with BPD in HK was namely antipsychotics, followed by antiepileptics (Figure 2a). Antipsychotics prescribing rose rapidly from 49.64% (95% CI 47.55-51.83) in 2001 to 66.35% (95% CI 64.25-68.51) in 2003 and then increased steadily to 72.89% (95% CI 71.44-74.37) in 2018. Quetiapine, olanzapine, risperidone, haloperidol, and chlorpromazine were the most frequently prescribed antipsychotics (Appendix 1a). Lithium prescribing increased from 20.09% (95% CI 18.77-21.49) in 2001 to 26.02% (95% CI 24.79-27.30) in 2004 and then dropped to 17.63% (95% CI 16.92-18.36) in 2018 as the least commonly prescribed treatment. Antipsychotics also remained as the most frequently prescribed monotherapy, followed by antiepileptics. The use of antidepressant monotherapy increased steadily to 3.42% (95% CI 3.12-3.76) in 2018 (Figure 2b).

##### 1.2 UK

Antidepressants prescribing remained steady from 46.90% (95% CI 45.26-48.59) in 2001 to 48.75% (95% CI 47.44-50.09) in 2018 as the most frequent drug class prescribed to patients with BPD in the UK, followed by antipsychotics, throughout the study period (Figure 2c). Antipsychotics prescribing increased from 36.71% (95% CI 35.27-38.21) in 2001 to 45.28% (95% CI 44.02-46.57) in 2018. The five most prescribed antipsychotics were quetiapine, olanzapine, aripiprazole, risperidone, and chlorpromazine, in order of prevalence (Appendix 1b). Lithium prescribing declined from 30.61% (95% CI 29.30-31.99) in 2001 to 16.03% (95% CI 15.29-16.81) in 2018. Particularly, antidepressant monotherapy prescribing decreased from 17.67% (95% CI 16.68-18.72) in 2001 to 15.62% (95% CI 14.89-16.38) in 2018 (Figure 2d).

#### 2 Mood stabilisers

##### 2.1 HK

By 2018, valproate (42.90%) was the most frequently prescribed mood stabiliser, followed by lithium (17.63%) (Figure 3a). Valproate prescribing increased from 16.97% (95% CI 15.77-18.27) in 2001 to 42.90% (95% CI 41.79-44.03) in 2018. The prevalence of lamotrigine prescribing increased from 0.17% (95% CI 0.08-0.35) in 2001 to 7.91% (95% CI 7.45-8.41) in 2018 while the use of carbamazepine showed a 4.30% reduction over the same period.

**Figure 4:**
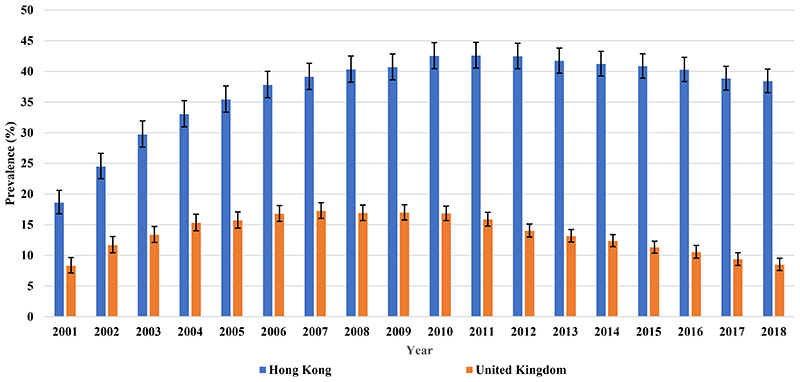
Prescribing of valproate to female patients of childbearing age (15-49 years) in Hong Kong and the United Kingdom

Valproate prescribing in women with BPD of childbearing age peaked at 42.58% (95% CI 40.54-44.73) in 2011 and then dropped to 38.41% (95% CI 36.53-40.38) in 2018 (Figure 4). As seen from the Sankey diagram (Figure 5a), a mean of 81.06% of valproate users each year remained on valproate over the study period. The proportion of patients who started valproate as their initial therapy increased from 28.64% in 2001 and reached a peak of 55.83% in 2012 then decreased gradually to 37.33% in 2018.

##### 2.2 UK

In spite of a rapid decline of lithium prescribing throughout the study period, lithium remained the most prescribed mood stabiliser with 16.03% (95% CI 15.29-16.81) of patients in 2018, followed by valproate (Figure 3b). Valproate prescribing more than doubled from 7.57% (95% CI 6.93-8.27) in 2001 to 17.81% (95% CI 17.13-18.51) in 2009, then reduced to 14.20% (95% CI 13.50-14.93) in 2018. The prevalence of lamotrigine prescribing increased from 0.49% (95% CI 0.35-0.70) in 2001 to 10.28% (95% CI 9.69-10.90) in 2018, while carbamazepine prescribing reduced by 7.27% over the same period.

Valproate prescribing in women with BPD of childbearing age doubled from 8.29% (95% CI 7.13-9.64) to 17.25% (95% CI 16.03-18.58) between 2001 and 2007; then dropped to 8.46% (95% CI 7.52-9.52) in 2018 (Figure 4). From Figure 5b, a mean of 72.98% of patients remained on valproate each year during the study period. The proportion of women who initially started valproate increased from 16.67% in 2001 and peaked at 23.20% in 2006, then dropped rapidly to 7.02% in 2018.

## Discussion

The overall prevalence of BPD diagnosis in HK and the UK increased from 2001 to 2018. This may reflect better awareness of mental illness and patients’ easier access to community psychiatric services for management ^32,33^. The prevalence of BPD in HK (0.066% in 2001; 0.183% in 2018) was similar to South Korea (0.11% in 2008; 0.20% in 2017) but both were almost two times lower when compared to the UK (0.150% in 2001; 0.357% in 2018) ^34^. In addition to a relatively more conservative attitude and stigma towards mental illness in Asian societies, earlier studies reported a positive association between the substance use disorders and BPD ^35–37^. According to the Global Burden of Disease Study 2016, the prevalence of substance use disorder in western countries was higher than in Asian countries ^38^. Therefore, a high prevalence of substance use disorder might be associated with the prevalence of BPD. In our study, more patients in the UK (11.81%) had concurrent alcohol and substance use disorders than in HK (7.98%). Furthermore, the median age at the first diagnosis of BPD in our cohorts was 38.8 years in HK and 43.65 years in the UK, which were consistent with other studies ^7,39,40^. Due to the nature of alternating episodes between mania and depression, some patients might experience depressive episodes before mania, leading to initial misdiagnosis of unipolar depression and hence resulting in a 10-year delay in treatment on average ^41^.

Our study findings reflected three major issues in clinical practice in HK and the UK: 1) underuse of lithium, 2) antidepressant monotherapy, and 3) overprescribing of valproate to women with childbearing potential.

We observed a substantial decline in patients treated with lithium in both HK and the UK from 2001 to 2018, alongside a simultaneous increase in prescribing of antipsychotics and antiepileptics. These findings were consistent with studies in other countries covering the period of 1995 to 2015 ^39,40,42^. The proportion of patients receiving lithium in HK (2001: 20.09%; 2018: 17.63%) and the UK (2001: 30.61%; 2018: 16.03%) was relatively lower compared to other countries (Denmark: from 37.5% in 1997 to 26.8% in 2012; Germany: from 44% in 2009 to 35.3% in 2018) ^39,43^. The rates of decline of lithium prescribing was faster in the UK than in HK and other countries (UK: 1.91-fold vs HK: 1.13-fold vs Denmark: 0.71-fold vs Germany: 0.80-fold), suggesting that not all new patients were offered lithium first-line in these countries, particularly in the UK ^39,43^. Currently, different international clinical guidelines recommend lithium, valproate, and antipsychotics (e.g. quetiapine, olanzapine) as first-line therapies for maintenance treatment ^13–16^. Therefore, it is reasonable that under-prescribing of lithium was observed in other countries as clinicians would prescribe alternative drugs other than lithium as recommended by the clinical guidelines. In 2014, the latest version of NICE guideline recommended lithium as the only first line maintenance treatment among all treatment alternatives, however the under-prescribing of lithium is more pronounced in the UK ^12^. Given there have been increasing studies that compared lithium with antipsychotics and/or antiepileptics, which suggested the superiority of lithium in relapse and suicide prevention, and hospitalisation ^44–47^, it is unclear why under-prescribing of lithium has still been emerging in clinical practice. Potential reasons restricting its use include 1) narrow therapeutic window and risk of toxicity, 2) complex regimen compared to antipsychotics and antiepileptics, 3) concerns on adverse effects on renal and thyroid functions and 4) non-adherence to therapeutic drug monitoring. Further studies will be warranted to understand the barriers of using lithium and encourage the prescribing of lithium unless contraindicated.

In our study, nearly 3% and 15% of patients with BPD received antidepressants alone in HK and the UK respectively by 2018. This has also been observed in other countries, such as Denmark, the US and Germany ^39,43,48^. Antidepressant monotherapy is not recommended in the clinical guidelines due to the risk of triggering manic episodes and lack of effectiveness in bipolar depression ^12–16,49,50^. The decreasing trend of antidepressant monotherapy in the UK reflects increased awareness of safety of antidepressant monotherapy by clinicians in the UK. Although antidepressant monotherapy was less common in HK, the increased use implicates the need to raise the awareness of local clinicians prescribing in concordance with this recommendation.

Concerns of the teratogenicity of valproate remain with accumulating evidence on the association of prenatal exposure of valproate in women of childbearing age and elevated risk of major congenital malformations and cognitive impairment ^11,51^. Our findings revealed 38.41% women with BPD of childbearing age were treated with valproate in HK in 2018, with majority initiating and remaining on valproate. This was much higher than other countries including the UK (8.47% in 2018) and Sweden (8% in 2013) which had reduced use of valproate in the same period ^52^.

Current regulations and policies in both regions might have an impact on valproate prescribing in women with childbearing potential. There is no standardised clinical guideline on BPD in HK so some local clinicians may base their drug choices on their clinical experience. Since 2014, the Pharmacy and Poison Board in HK has only labelled all valproate products with precautionary teratogenic warnings, while the Medicines and Healthcare Products Regulatory Agency in the UK implemented a regulatory measure in March 2018, to advise against clinicians prescribing valproate to women of childbearing potential and ensuring patients clearly understand the risk and benefits of treatment and effective contraception must have taken place if valproate is the only available option ^53,54^. Impulsivity and hypersexuality during mania empirically increase the risk of unprotected sex and therefore unplanned pregnancies ^55^. Foetal valproate exposure during unplanned pregnancies could lead to higher incidence of foetal malformations and potentially higher abortion rates in patients with BPD. Overprescribing of valproate to women is a highly important public health issue, especially in HK so there is an urgent need to promptly review current treatment guidelines and regulatory measures to restrict the general use of valproate in women with BPD and advise clinicians to consider alternative drug choices as initial treatment where applicable.

There are some limitations in this study. CDARS captures clinical data only from the public healthcare system in HK, so data from the private practice is not available and the actual prevalence might be underestimated. Patients with higher socio-economic status might seek consultation and treatment from the physicians at private sector but patients with BPD usually require lifelong treatment and would often prefer public services due to subsidised medical costs ^56^. As aforementioned, majority of the psychiatric patients utilised mental health services provided by the public healthcare sector ^19^. On the other hand, IMRD captures the clinical information in primary care sectors. Patients with mental disorders in the UK are usually diagnosed in secondary care and then fed back to general practitioners in primary care. Therefore, data used in this study is likely to include most of the patients with BPD in two regions and the actual prevalence will not be largely affected. Furthermore, there might be some potential heterogeneities in both CDARS and IMRD databases, e.g. coding practices but the estimations were based on clinical data within each database so the heterogeneities would not affect the estimated trends in two regions and the conclusion of this study.

## Conclusion

The increasing prevalence of BPD in HK and the UK may reflect improved awareness of mental disorders and better resources for psychiatric services for management. Our findings suggested some important trends in BPD treatment should be further explored, particularly, underuse of lithium, antidepressant monotherapy and overprescribing of valproate to women of childbearing age. Regular review of local treatment guidelines and regulations of prescribing practice should be promptly implemented to ensure the safety of medication use in patients with BPD.

## Supplementary Material

Appendix A1

Appendix A2

## Figures and Tables

**Figure 1a:**
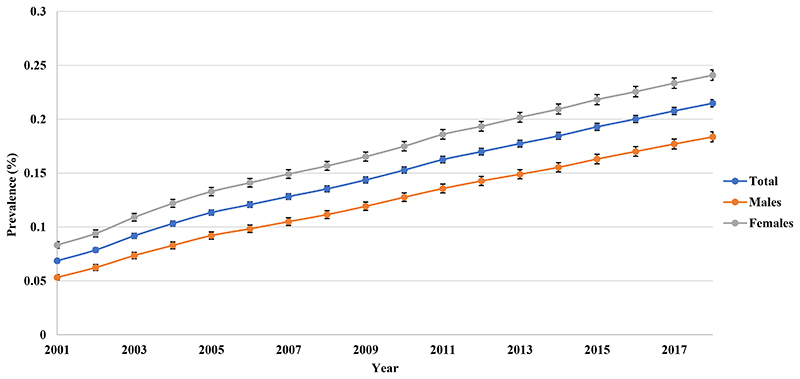
Annual prevalence of bipolar disorder in Hong Kong stratified by sex

**Figure 1b:**
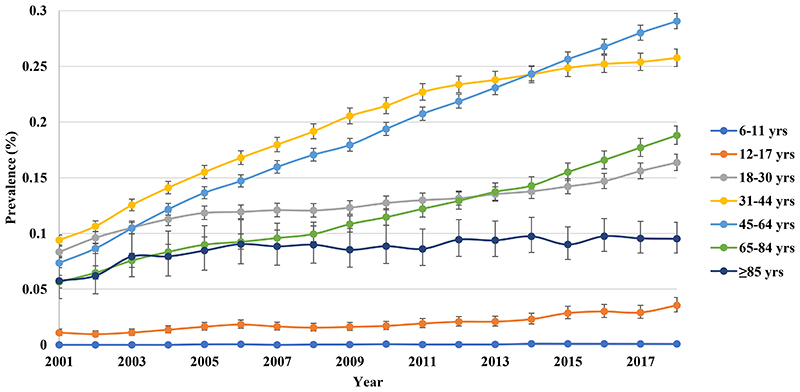
Annual prevalence of bipolar disorder in Hong Kong stratified by age groups

**Figure 1c:**
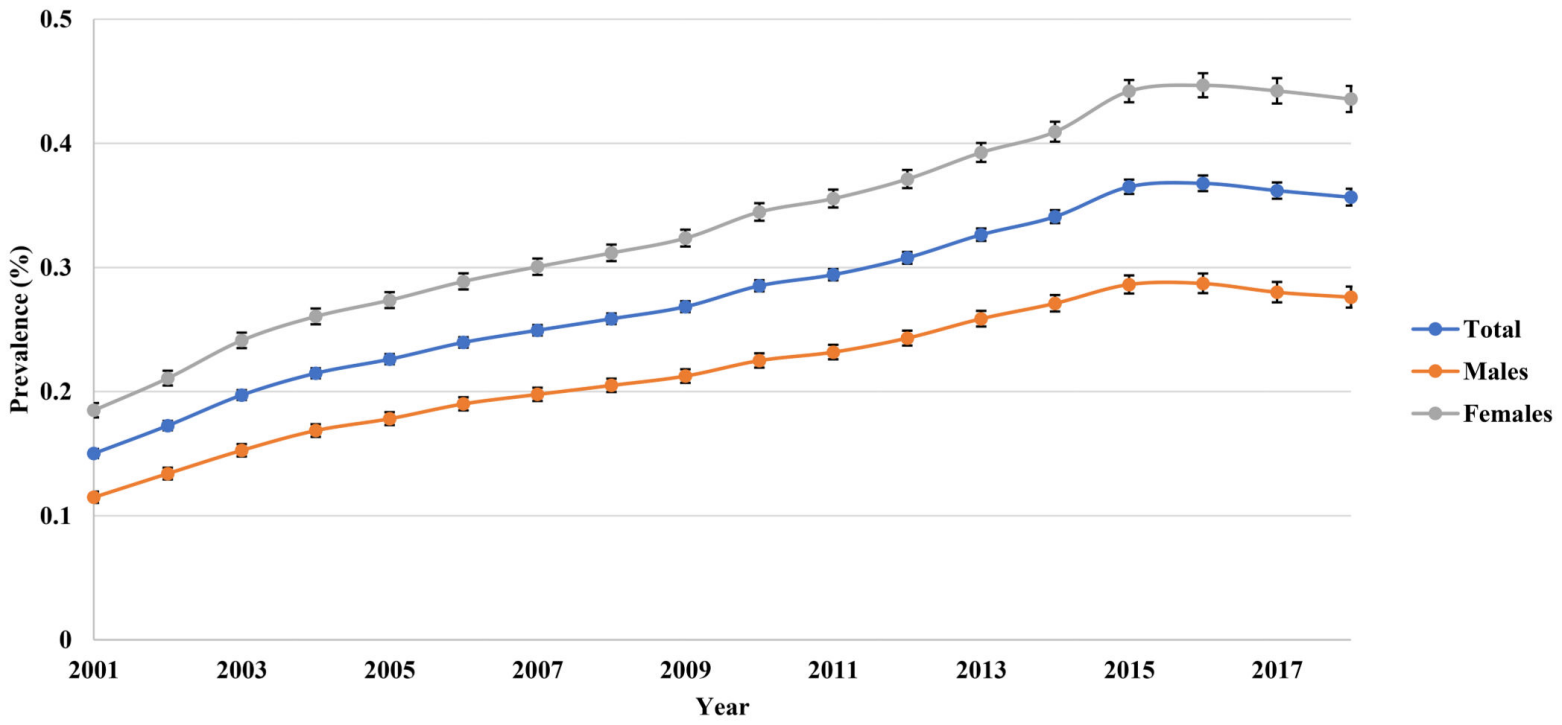
Annual prevalence of bipolar disorder in the United Kingdom stratified by sex

**Figure 1d:**
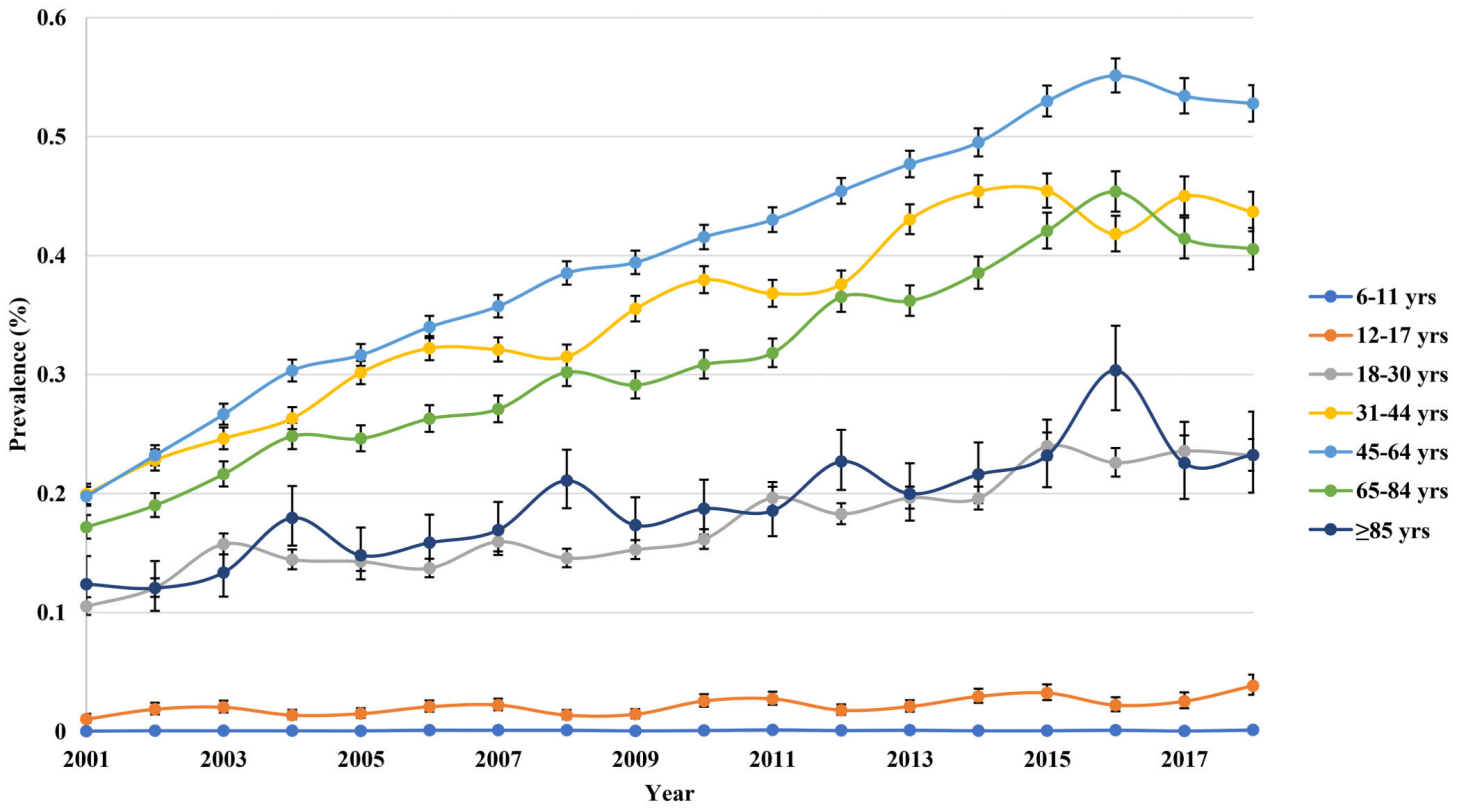
Annual prevalence of bipolar disorder in the United Kingdom stratified by age groups

**Figure 2a:**
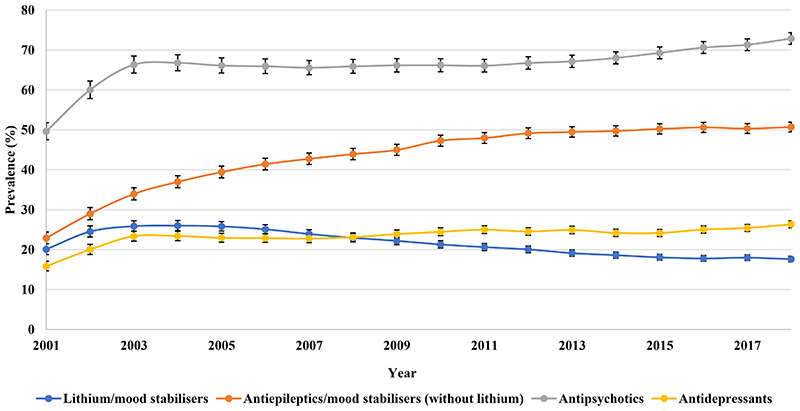
Annual prevalence of psychotropic drug users in Hong Kong

**Figure 2b:**
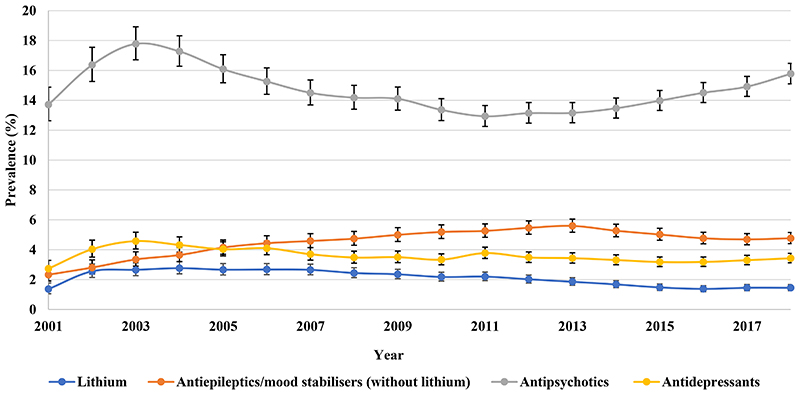
Prevalence of monotherapy of different drug classes in Hong Kong

**Figure 2c:**
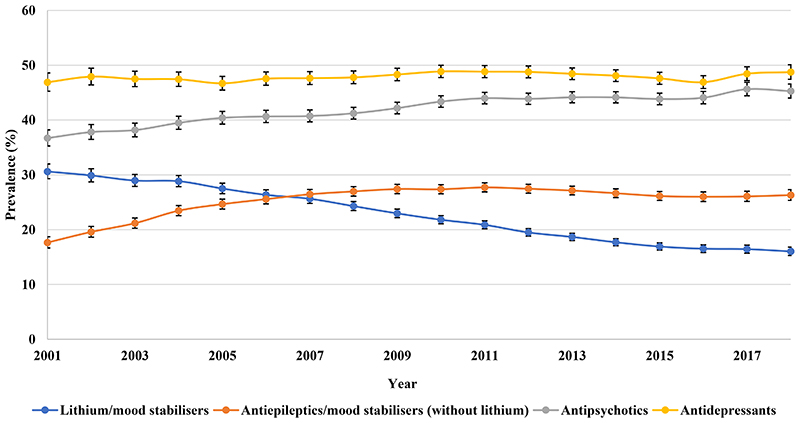
Annual prevalence of psychotropic drug users in the United Kingdom

**Figure 2d:**
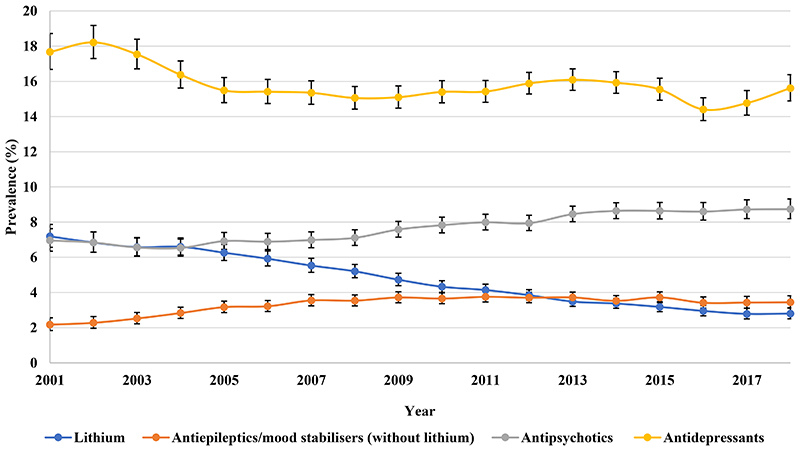
Prevalence of monotherapy of different drug classes in the United Kingdom

**Figure 3a:**
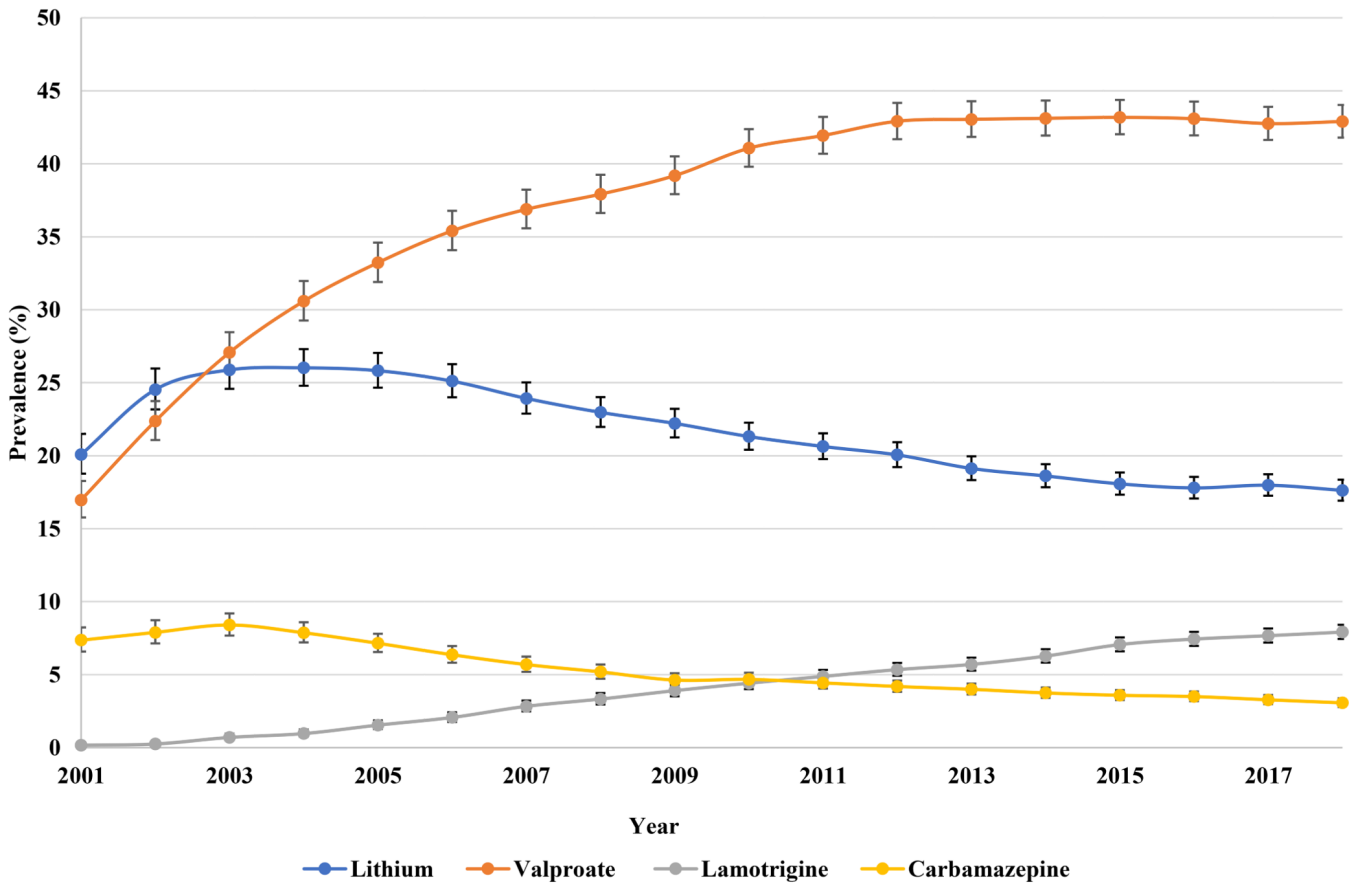
Prescribing of individual mood stabilisers in Hong Kong

**Figure 3b:**
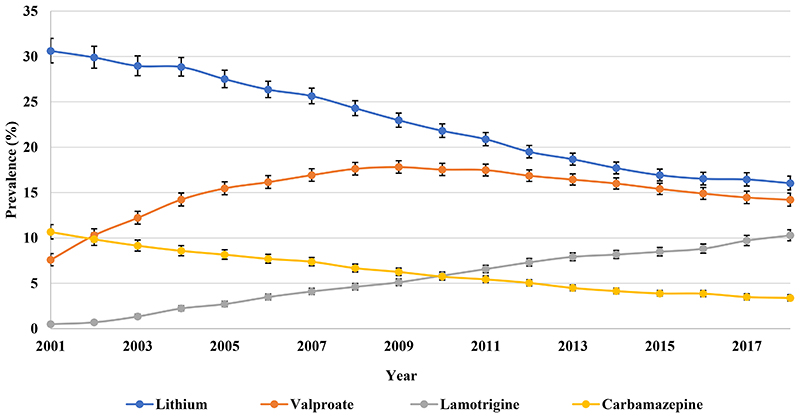
Prescribing of individual mood stabilisers in the United Kingdom

**Figure 5a:**
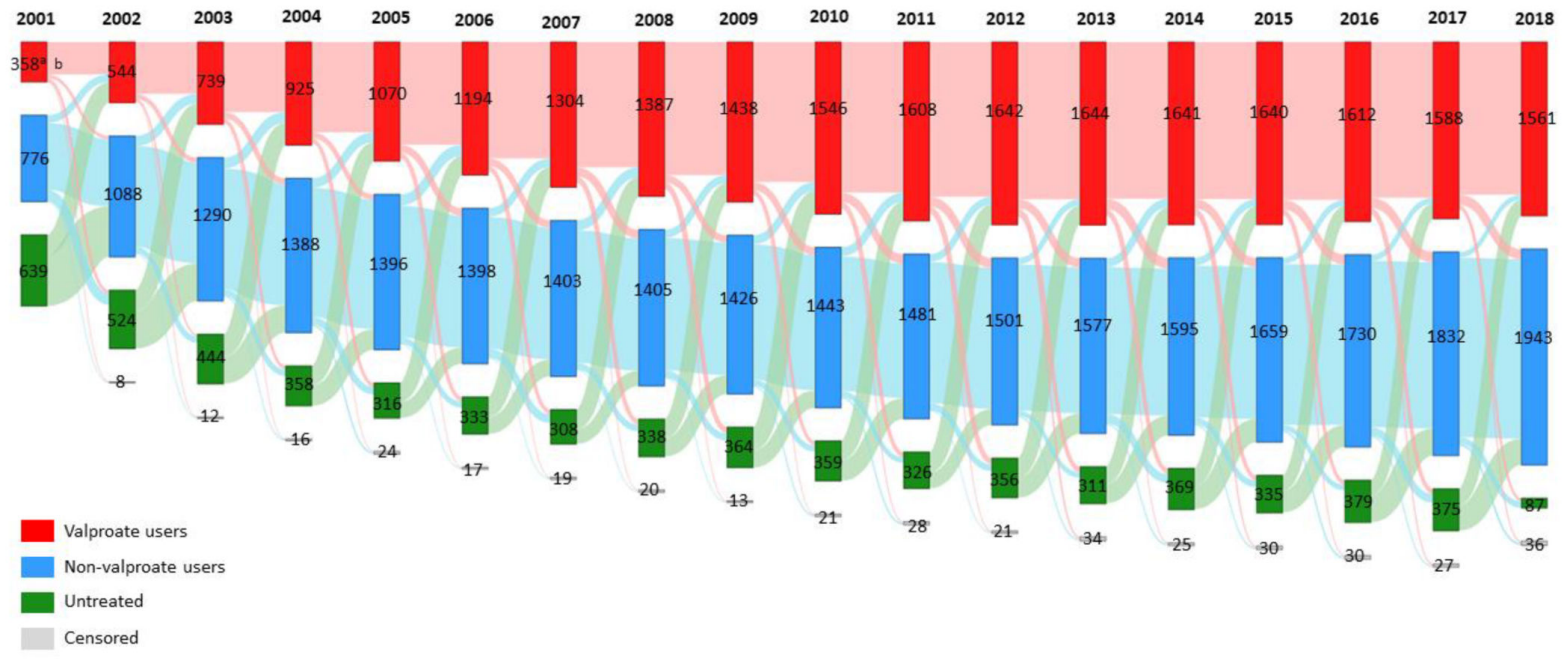
Treatment pathways of female patients of reproductive age (15-49 years) in Hong Kong depicted by a Sankey Diagram ^a^The number inside the bar indicates the number of people who belongs to respective categories. ^b^The heights of the shades between two bars are proportional to proportion of people who remained, switched to other categories or were censored from the database. Censored: patients who died or left the database within that particular year

**Figure 5b:**
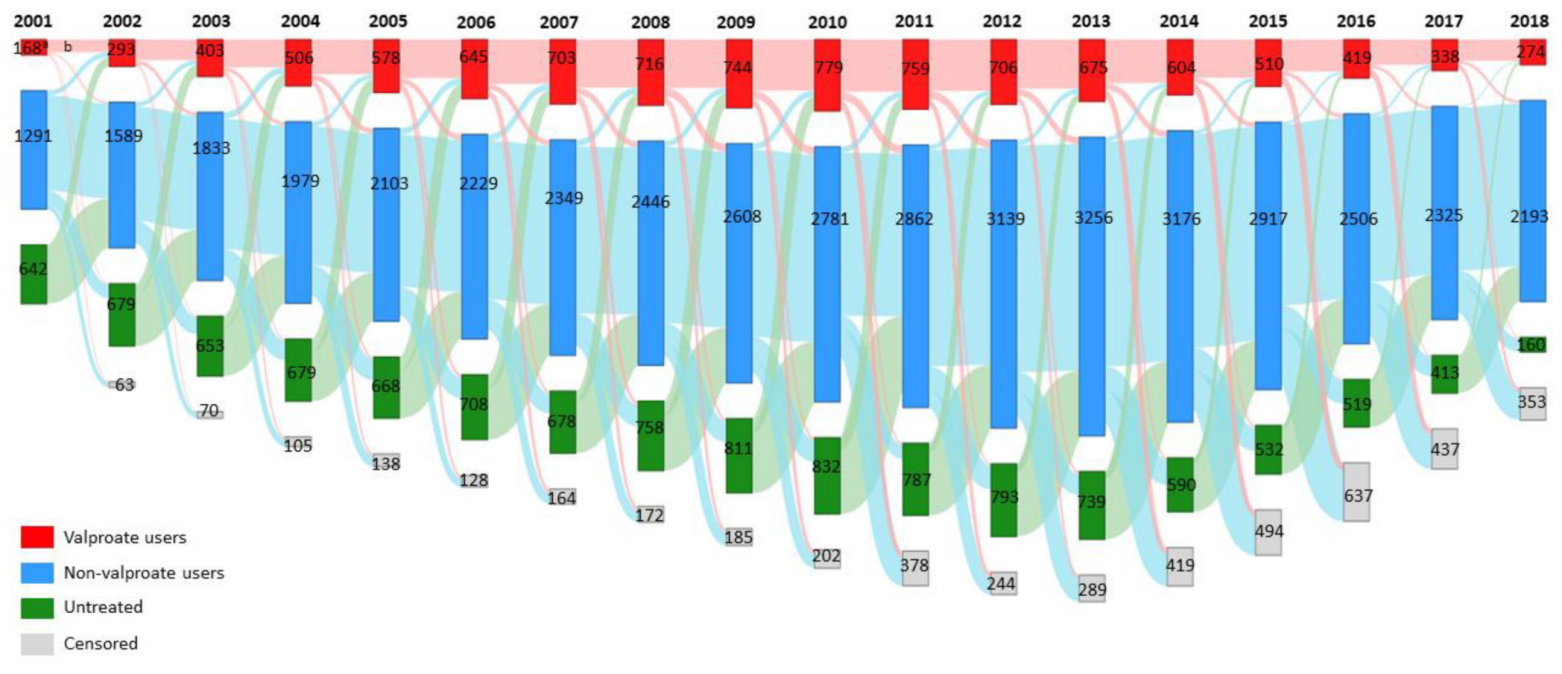
Treatment pathways of female patients of reproductive age (15-49 years) in the United Kingdom depicted by a Sankey Diagram ^a^The number inside the bar indicates the number of people who belongs to respective categories. ^b^The heights of the shades between two bars are proportional to proportion of people who remained, switched to other categories or were censored from the database. Censored: patients who died or left the database within that particular year

**Table 1:** Characteristics of treated and untreated patients with bipolar disorder in Hong Kong and the United Kingdom

	CDARS (HK)			IMRD (UK)		
Characteristics	Treated	Untreated^†^	Total	Treated	Untreated^†^	Total
N	15287	1960	17247	30140	6047	36187
**Gender**						
Male (%)	6042 (39.52)	853 (43.52)	6895 (39.98)	11632 (38.59)	2788 (46.11)	14420 (39.85)
Female (%)	9245 (60.48)	1107 (56.48)	10352 (60.02)	18508 (61.41)	3259 (53.89)	21767 (60.15)
Age at first diagnosis, median (IQR)	38.92 (22.46)	37.38 (23.12)	38.8 (22.56)	44.18 (26.69)	40.54 (28.64)	43.65 (24.59)
**Age distribution, n (%)**						
6-11 years	13 (0.09)	6 (0.31)	19 (0.11)	11 (0.04)	20 (0.33)	31 (0.09)
12-17 years	690 (4.51)	58 (2.96)	748 (4.34)	366 (1.29)	201 (3.32)	567 (1.57)
18-30 years	4136 (27.06)	610 (31.12)	4746 (27.52)	5757 (19.10)	1644 (27.19)	7401 (20.45)
31-44 years	4949 (32.37)	605 (30.87)	5554 (32.20)	9507 (31.54)	1660 (27.45)	11167 (30.86)
45-64 years	4302 (28.14)	469 (23.93)	4771 (27.66)	10020 (33.24)	1502 (24.84)	11522 (31.84)
65-84 years	1119 (7.32)	192 (9.80)	1331 (7.72)	4082 (13.54)	853 (14.11)	4935 (13.64)
85 years or above	78 (0.51)	21 (1.02)	98 (0.57)	397 (1.32)	167 (2.76)	564 (1.56)
**Mental Comorbidities, n (%)**						
ADHD	49 (0.32)	9 (0.46)	58 (0.31)	407 (1.37)	65 (1.07)	472 (1.30)
Alcohol and substance use disorder	1240 (8.11)	137 (6.99)	1377 (7.98)	3781 (12.54)	493 (8.15)	4274 (11.81)
Anxiety disorder	1959 (12.81)	129 (6.58)	2088 (12.11)	12340 (40.94)	1494 (24.71)	13834 (38.23)
Epilepsy	422 (2.76)	33 (1.68)	455 (2.64)	1138 (3.78)	120 (1.98)	1258 (3.48)
Personality disorder	1012 (6.62)	53 (2.70)	1065 (6.17)	3075 (10.20)	394 (6.52)	3469 (9.59)
Psychosis	3737 (24.45)	334 (17.04)	4071 (23.60)	4607 (15.06)	689 (11.39)	5296 (14.64)
Schizophrenia	4023 (26.32)	181 (9.23)	4204 (24.38)	3213 (10.22)	427 (7.06)	3640 (10.06)

† Untreated: patients received <2 prescriptions of any study medications during the study period; Abbreviations: ADHD Attention Deficit Hyperactivity Disorder, HK Hong Kong, IQR Interquartile range, SD Standard deviation, UK United Kingdom

## Data Availability

The data that support the findings of this study are available on request from the corresponding author. The data are not publicly available due to privacy or ethical restrictions.

## Key points

- Increasing prevalence of bipolar disorder in Hong Kong and the United Kingdom might reflect improved awareness and positive attitude towards treatment.
- Overprescribing of valproate to women with bipolar disorder with childbearing age has been more alarming in Hong Kong compared to other countries, against best advice from international clinical guidelines.
- Under-prescribing of lithium was observed in Hong Kong and other western countries. The rate of decline of lithium use was more notable in the United Kingdom.
- Proportion of patients with bipolar disorder receiving antidepressant monotherapy was 4.6 times more in the United Kingdom than in Hong Kong (15.62% vs 3.42%).
- Review of current clinical treatment guidelines and regulations of prescribing practice by local clinicians should be immediately implemented to ensure the safe use of medications in patients with bipolar disorder
