# Supplementary material for: Bipolar disorder prevalence and psychotropic medication utilisation in Hong Kong and the United Kingdom
Source: Pharmacoepidemiol Drug Saf. Author manuscript; Available in PMC 2022 Jul 20. (PMC7613092; doi:10.1002/pds.5318)
Supplement: Appendix A1 [file EMS150066-supplement-Appendix_A1.pptx]

## Slide 1
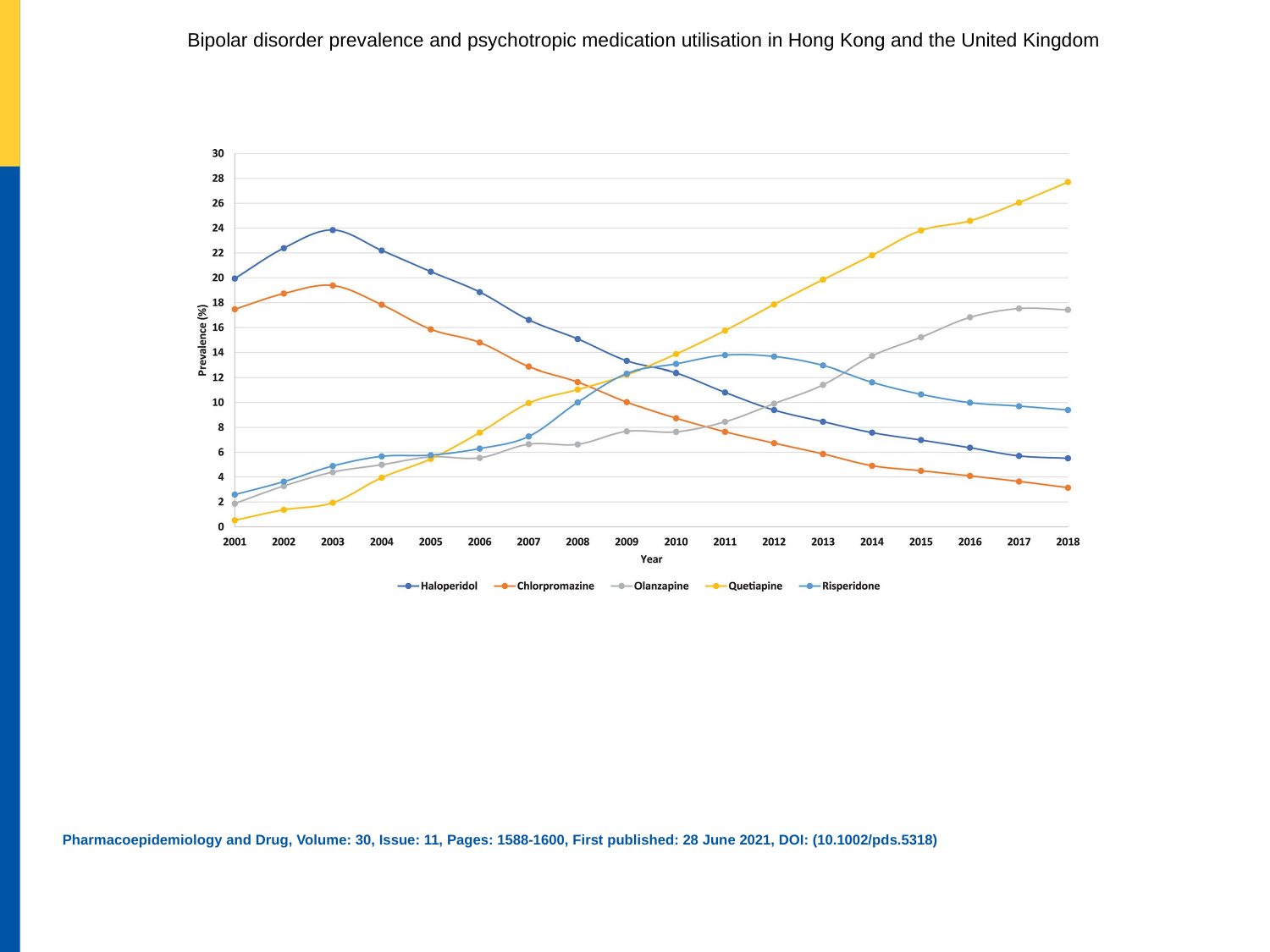

Bipolar disorder prevalence and psychotropic medication utilisation in Hong Kong and the United Kingdom
Pharmacoepidemiology and Drug, Volume: 30, Issue: 11, Pages: 1588-1600, First published: 28 June 2021, DOI: (10.1002/pds.5318)
